# [Retracted] microRNA‑196b promotes cell migration and invasion by targeting FOXP2 in hepatocellular carcinoma

**DOI:** 10.3892/or.2023.8660

**Published:** 2023-11-06

**Authors:** Zhaoxiang Yu, Xiaobo Lin, Ming Tian, Weiping Chang

Oncol Rep 39: 731–738, 2018; DOI: 10.3892/or.2017.6130

Following the publication of the above paper, it was drawn to the Editor's attention by a concerned reader that the images showing metastatic nodules in the mouse lung in Fig. 4 on p. 735 had apparently previously been published a few years earlier in the following paper: Jia D, Yan M, Wang X, Hao X, Liang L, Liu L, Kong H, He X, Li J and Yao M: Development of a highly metastatic model that reveals a crucial role of fibronectin in lung cancer cell migration and invasion. BMC Cancer 10: 364, 2010. Moreover, among the cell migration and invasion assay data shown in Figs. 3B and D, and 6B and 7B, there were a number of overlaps noted among the data panels such that data which were intended to show results obtained under different experimental conditions were likely to have been derived from a smaller number of original sources.

Owing to the fact that the contentious data in the above article had already been publihed prior to its submission to *Oncology Reports*, in addition the other features of concern regarding the data, the Editor has decided that this paper should be retracted from the Journal. The authors were asked for an explanation to account for these concerns, but the Editorial Office did not receive a reply. The Editor apologizes to the readership for any inconvenience caused.

